# *Aedes albopictus* Adult Medium Mass Rearing for SIT Program Development

**DOI:** 10.3390/insects10080246

**Published:** 2019-08-09

**Authors:** David Damiens, Lucie Marquereau, Cyrille Lebon, Gilbert Le Goff, Benjamin Gaudillat, Nausicaa Habchi-Hanriot, Louis-Clément Gouagna

**Affiliations:** 1 Institut de Recherche pour le Développement (IRD), UMR MIVEGEC (CNRS/IRD/Université de Montpellier), Maladies Infectieuses et Vecteurs, Ecologie, Génétique, Evolution et Contrôle, 34394 Montpellier, France; 2 IRD Réunion/GIP CYROI (Recherche Santé Bio-innovation), 97491 Sainte Clotilde, Reunion Island, France

**Keywords:** mosquito facility, insect production, male mosquito, sterile insect technique

## Abstract

For the production of several hundred thousands of *Aedes albopictus* sterile males for the implementation of a Sterile Insect Technique (SIT) program, no costly mass-rearing equipment is needed during the initial phases, as optimized rearing at laboratory scale can be sufficient for the first steps. The aim of this study was to maximize the egg production by optimizing adult rearing methods for *Ae. albopictus*. The effect of parameters such as male/female ratio, density of adults, membrane type for blood feeding, quantity of blood delivered, continuous or discontinuous blood feeding, and surface of substrates for egg laying on overall egg production was tested to find optimized conditions. Based on the number of eggs produced per cage in response to the parameters tested, the optimum cage set-up was seen to be 1500 adults in a 30 × 30 × 30 cm cage with a male/female sex ratio of 1:3, fed by fresh bovine blood for periods of 30 min using a cellulose membrane covering a 10 cm stainless steel plate heated by a Hemotek device, and the provision of five oviposition cups to collect eggs. With this set-up, production per cage can reach a maximum of 35,000 eggs per week.

## 1. Introduction

The Sterile Insect Technique (SIT) [1] is a biological control method used to control insect pests by releasing a large number of sterile males into wild populations. These will compete with wild males to mate with females in the field and thereby reduce the fertility of the target population. The success of the SIT depends mainly on a continuous release of overflooding numbers of sterile males compared to wild males within the target area [2,3] which could require several million per week in an area-wide suppression program. To enable such a high production of insects, mass-rearing equipment and suitable rearing methods have to be used as have been developed for *Aedes* spp. control programs including a larval rearing unit [4,5], adult mass-rearing cage [6,7], and mass-rearing methodology including egg management [8,9] or larval diet development [10].

The high initial investment for infrastructure, materials, and human resources to run an effective mass-rearing facility [11] could generate an economic barrier to a number of countries and is likely to inhibit the launching of an SIT program even before the feasibility assessment or pilot trial. However, for the first steps of an SIT program, the production of millions of males is not required. Depending on available resources, the size of a pilot study can be adjusted to the production capacity and fewer numbers are needed for small-scale pilot suppression trials. Following the selection of suitable field sites, various study designs are needed to assess population characteristics of the target species, such as its spatial and temporal distribution and dynamics [12,13], as well as its dispersal range [14] and population size [15,16], which can be done by mark–release–recapture (MRR) studies. With the information attained, a small-scale pilot suppression trial can be planned and executed accordingly to test parameters such as males release ratios needed or swath width before initiating an extensive suppression program [17]. Generally, in such first release trials, a release of some tens of thousands of sterile insects per week for several months can be enough. The production of this quantity of males does not necessarily require mass-rearing equipment but does need optimal management of regular laboratory rearing space. A first step to managing laboratory rearing space may be to maximize insect density and handling in rearing cages in an attempt to optimize egg-to-adult production without negatively affecting the quality of reared individuals. Using available resources, our efforts in the present study were to develop an efficient medium-scale, laboratory production system useful for a small-scale pilot demonstration of SIT against *Aedes albopictus* (Skuse) in La Reunion. The effects of various parameters such as male/female cage ratio, density of adults, membrane type for blood feeding, quantity of blood offered, continuous or discontinuous blood-feeding periods, and surface substrates for egg laying on overall egg production were tested here, resulting in a proposal for optimized cage set-up based on attained egg production data.

## 2. Materials and Methods

### 2.1. Mosquito Rearing, Maintenance, and Blood Feeding

All mosquitoes used in the present study were originated from a colony originally established from eggs collected in the field at Saint Marie in 2014. All experiments described herein were carried out in a climate-controlled insectary (T: 27 ± 2 °C, RH: 75 ± 2%, light: 12L:12D), where two-day-old adult *Aedes albopictus* were placed in standard rearing cages (30 × 30 × 30 cm, Bugdorm, Taiwan) with continuous access to 5% (wt:vol) sucrose solution. The first blood meal was offered 7 days after emergence to allow both reproductive maturation and mating. Every week, blood meals were offered to females for 30 min (or two times 15 min for experiment 5) on Monday, Tuesday, Thursday, and Friday. Before blood meals, females were deprived of sugar for 5 h. The blood was delivered with a Hemotek system [18] with an aluminum blood plate (10 cm diameter, 1 cm thickness, and 20 mL total blood capacity or 15 cm (50 mL of blood) in experiment 4). Defibrinated fresh bovine blood stored at 4 °C gradually brought to room temperature was injected between the plate and a membrane attached to the plate. Egg collection took place twice a week by placing three oviposition cups (excepted in experiment 6) consisting of a piece of filter paper (180 × 70mm) on the side of a cup filled with 30 mL of water, placed for 24 h on Monday and Thursday. Egg production (number of eggs on the filter paper counted under a stereomicroscope) and adult mortality (daily number of dead males and females in each rearing cage) were recorded.

The experiments described below were successively carried out on separate occasions, being designed to examine the suitability of parameters such as rearing density, male-biased sex ratio, blood-feeding surface and regimen, and egg-laying substrate for efficient egg production in small cages.

### 2.2. Experiment 1: Effect of Sex Ratio

To optimize the number of females in rearing cages, the effect of three experimental ratios of males to females (1:1, 1:3, and 1:5) on egg production was tested (three replicates each, one replicate represents one cage with 1000 adults). For a total of 1000 adults per cage, 500 males and 500 females, 750 males and 250 females, and finally 833 males and 177 females were added to cages for the ratios 1:1, 3:1, and 5:1, respectively.

### 2.3. Experiment 2: Effect of Adult Density

Three adult densities were tested: 1000, 1500, and 2000 adults in 30 × 30 × 30 cm cages. The effect of adult numbers in cages on egg production and adult survival was observed. A ratio of 1:3 (male:female) was chosen for further studies. Three cages per density were assessed.

### 2.4. Experiment 3: Effect of Blood Delivery

Three types of membranes for blood feeding were tested in this experiment (three replicates each, one replicate represents one cage with 1500 adults with a male:female ratio of 1:3): turkey-skin membrane, Parafilm, and collagen membrane (Discovery Workshops). After collection, turkey skin was cleaned with deionized water and stored at −18 °C until used. Before blood feeding, the membrane was brought to ambient temperature by removing it from the freezer one hour before the feeding. The skin was then placed on the Hemotek plate and held in place with a rubber band. For the Parafilm, pieces were cut just before the experiment. Two pieces were stretched, tightened, and sealed to one plate to avoid leakage. One piece of collagen membrane was cut and placed on the plate secured by a rubber band. After feeding, egg production was monitored for 35 days during which adult survival was recorded.

### 2.5. Experiment 4: Effect of Blood Quantity/Plate Surface

Provisioning females with a large feeding surface could increase feeding rate, leading to an increase of egg production. Two sizes of plates for blood feeding were tested in this experiment (three replicates each, one replicate was one cage with 1500 adults with a male:female ratio of 1:3 and collagen membrane): 10 cm and 15 cm diameter. One piece of collagen membrane was cut to appropriate size and placed on each plate secured by a rubber band. To fill the plate chamber (between the plate and the collagen membrane), different quantities of blood were added according to the plate size: 20 mL for the 10 cm plate and 50 mL for the 15 cm plate. Subsequent egg production was recorded for 35 days.

### 2.6. Experiment 5: Effect of Continuous or Discontinuous Blood-Feeding Periods

During the previous experiments, it was observed that although more females could be quickly attracted when the plate was placed on the cage, only a few of them were observed taking blood meals several minutes later. The appearance of the warm plate was seen to act as a better stimulant compared to the long presence of the plate. To test this hypothesis, two procedures for blood feeding were tested in this experiment (three replicates each, one replicate was one cage with 1500 adults with a male:female ratio of 3:1 and collagen membrane, 10 cm plate) with the same duration of blood feeding but one with a continuous period (i.e., 30 min at once) and one with a fragmented period (i.e., 30 min divided into two periods of 15 min with a 30 min break). Blood was prepared as described above. Subsequently, egg production was recorded for 35 days.

### 2.7. Experiment 6: Effect of Substrate Surface for Egg Laying

According to the number of females ready to lay eggs, the surface available should be greater than the quantity of eggs that these females are expected to lay. To determine the surface requirements, three set-ups were tested (three replicates each, one replicate was one cage with 1500 adults with a male:female ratio of 1:3 and collagen membrane, 10 cm plate, continuous feeding) with one, three, and five oviposition cups available in the cages. Blood was prepared as described above. Egg production was recorded for 35 days.

### 2.8. Statistical Analysis

Data was analyzed using Minitab 16 [19]. Adult survival curves were estimated using the Kaplan-Meier method. The difference in survival between treatments was compared using the log-rank test. A one-way ANOVA and student *t* test were used to assess the difference between the mean total numbers of eggs produced by cages following different treatments. Before using ANOVA tests, the normality of each data set was tested with the Shapiro–Wilk Normality Test and the distributions were shown to be normal.

## 3. Results

### 3.1. Effect of Sex Ratio

Although there was a trend of increasing egg production with the increase of sex ratios, (25,078 ± 4539, 35,202 ± 9236, and 38,196 ± 7532 mean eggs produced in total in cages with ratios (male:female) of 1:1, 1:3, and 1:5, respectively), there was no significant effect of ratio on egg production (ANOVA, F_2,6_ = 2.61, *p* = 0.153).

### 3.2. Effect of Adult Density

**Figure 1:**
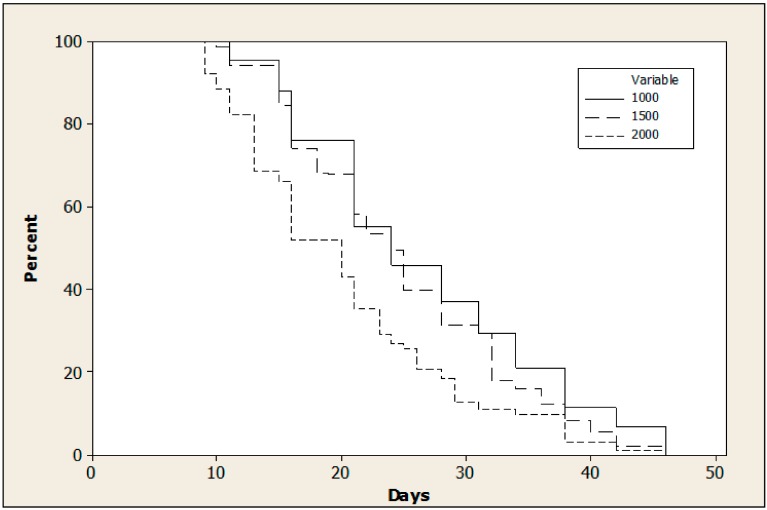
Survival curves of adults according to three different cage densities: 1000, 1500, and 2000 adults with a ratio of one male for three females in standard rearing cages (30 × 30 × 30 cm) with continuous access to 5% (wt:vol) sucrose solution.

Density of *Ae. albopictus* adults had no significant effect on egg production (ANOVA, F_2,6_ = 0.81, *p* = 0.49). The mean number of eggs collected was 30,598 ± 4852, 40,077 ± 8864, and 40,816 ± 16,144 per cage for densities of 1000, 1500, and 2000 adults, respectively. The survival curves of adults was significantly influenced by the adult density (Figure 1, log rank test, Chi^2^ = 217.049, df = 2, *p* < 0.001).

### 3.3. Effect of Blood-Feeding Membrane

**Figure 2:**
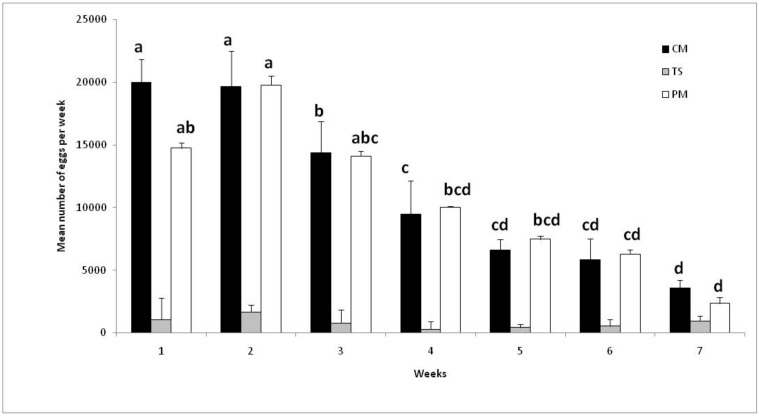
Number of eggs produced by cages fed with cellulose membrane (CM), turkey skin (TS), and Parafilm^®^ membrane (PM). Histograms with the same letter indicate that the results are not significantly different (*p* > 0.05, Tukey’s HSD post hoc test following an ANOVA procedure within each treatment). No letters indicate no significant difference between the treatments.

No impact of different blood-feeding membranes on the *Ae. albopictus* adult survival was observed (log rank test, Chi^2^ = 1.1630, df = 2, *p* = 0.56). However, the type of blood-feeding membrane had a significant effect on total egg production per cage (ANOVA, F_2,7_ = 100.4, *p* < 0.001). Post hoc tests indicate that feeding mosquitoes with Parafilm® or cellulose membrane had no significant effect on egg production per cage (79,515 ± 5457 and 74,782 ± 13,018 eggs, respectively). The cages fed with turkey skin produced a significantly lower quantity of eggs (5689 ± 2572) compared to Parafilm® and cellulose membranes. Figure 2 shows the evolution of the mean production of eggs per cage and per week over seven weeks. For the cellulose membranes, the production stayed constant over the three first weeks and decreased thereafter. For the Parafilm membranes, the evolution was similar with a peak in the second week. These results can help determine the period over which a cage should be maintained and how it can be best utilized.

### 3.4. Effect of Blood Quantity/Plate Surface

When compared, the mean total number of eggs produced by cages fed on blood through a 10 cm or 15 cm diameter plate showed no significant difference (86,396 ± 15,580 eggs and 89,536 ± 5832, respectively, *t* test, *t* = 0.3269, df = 4, *p* = 0.76). Moreover, a higher feeding surface area did not attract more females to blood-feed and lay eggs since there was no significant difference in the egg production between the two plate sizes following the first four blood meals (56,891 ± 15,312 eggs for 10 cm and 62,417 ± 10,483 for 15 cm, *t* test, *t* = 0.5152, df = 4, *p* = 0.63).

### 3.5. Effect of Continuous or Discontinuous Blood-Feeding Period

The mean total number of eggs produced by cages with continuous and discontinuous blood feeding was 95,188 ± 19,728 eggs and 89,653 ± 3096, respectively. There was no significant difference between the two treatments (*t* test, *t* = 0.4801, df = 4, *p* = 0.66).

### 3.6. Effect of Substrate Surface Available for Egg Laying

Increasing the number of oviposition substrates in the rearing cage did not lead to an increased egg production by females (ANOVA, F_2,6_ = 4.4, *p* = 0.7) with 52,249 ± 9026, 72,996 ± 7341, and 64,918 ± 9454 eggs for 1, 3, and 5 papers, respectively.

## 4. Discussion

Using a standard 30 × 30 × 30 cm cage, several parameters such as male:female sex ratio, density of adults, membrane type for blood feeding, quantity of blood offered, continuous or discontinuous blood-feeding regimen, and a range of egg-laying surfaces were separately manipulated to reach a more efficient rearing set-up for increased *Aedes albopictus* egg production. According to the parameters tested, the optimum cage set-up seems to be 1500 adults in a 30 × 30 × 30 cm cage with a male:female sex ratio of 1:3, fed by fresh bovine blood for a duration of 30 min with cellulose membrane on a 10 cm plate, and with three oviposition cups to collect eggs.

With this set-up, egg production per cage can reach a maximum of 35,000 eggs per week. For the same ratio but with 4000 *Ae. albopictus* adults in 30 × 30 × 30 cm cages, Zhang et al. [20] observed a production of 142,000 after two blood feedings. It is difficult to hypothesize about the difference in number of eggs per female produced by females in the current study (28 eggs) and the females from Zhang et al. [20] (47 eggs) due to differences in rearing conditions. Difference in strains, blood quality, and blood delivery could be some of the parameters that could influence the egg production. For *Aedes aegypti* (Linnaeus), Imam et al. [21] suggest the introduction of 2000 pupae in a 30 × 30 × 30 cm cage with a ratio of 3 females to 1 male. However, no result in egg production was associated with this statement. In Carvalho et al. [22], one 30 × 30 × 30 cm cage of *Aedes aegypti* with 1000 males and 3000 females produced an average 143,000 eggs per week with two blood feedings per week. Adults of *Ae. aegypti* can live in more crowded conditions than *Ae. albopictus* and females lay almost three times more eggs.

In our conditions, there is a trend of increasing egg production according to the male:female ratio. This is in concordance with the results observed by Zhang et al. [20] in small cages with a 37.9% higher egg production with a 3:1 ratio compared to a 1:1 ratio. Since *Ae. albopictus* males are able to copulate efficiently in rapid succession with 5 females in one day and 11 females during his life [23], a low ratio of males would still result in a high number of inseminated females in cages, leading possibly to an increase in the production of eggs per cage. The ratio of 1:5 could have been chosen since the production is similar to the 1:3 ratio, but the management of pupae needed to achieve this ratio is unrealistic under the routine laboratory rearing system.

Generally, when insect-rearing conditions become more crowded, their survival and fecundity usually decrease [24]. In our conditions, the overcrowding of *Ae. albopictus* cage populations seem to be limited at 1500 to 2000 adults for our strain. It is a lower density compared to the one tested by Zhang et al. [20], who were able to maintain a 30 × 30 × 30 cm cage with 4000 individuals for two blood feedings. However, for Zhang et al. [20], no data on mortality is available, but the production seems consistent with good survival since the cages were able to produce 142,000 eggs for this period versus two blood feedings in 10 days. Differences between strains could explain such intraspecific variation in life history traits such as larval fitness [25,26] or adult fitness [27,28]. Moreover, artificial selection associated with rearing conditions could result in behavioral changes [29,30]. Moreover, in Zhang et al. [20], it was a triple Wolbachia-infected HC strain which was generated by the transfer of the wPip strain through embryonic cytoplasmic injections into an *Ae. albopictus* Houston strain.

For the different membranes used for blood feeding, results showed that there was no effect on egg production in *Ae. albopictus* females following the use of Parafilm^®^ or cellulose membranes. However, the manipulation of cellulose membranes is easier and has a very low probability of leaking or tearing contrarily to Parafilm. In addition, the increase of surface area of blood available by changing the diameter of the plate did not increase the cage egg production. However, for the mass-rearing cage where the number of females is higher, the use of bigger plates should be tested. The cellulose membrane seemed to be optimum for the feeding on the Hemotek device and could probably be used on some of the numerous blood-feeding devices developed in different laboratories or facilities [31] that could be cheaper than the Hemotek device. No effect of the frequency (one time 30 min versus two times 15 min) was observed on the egg production. When the plate is placed on the cage, among the parameters that usually attract the females to the host (including carbon dioxide, water vapor, and host odors) [32], it is probably the warm blood that mimics host body heat. Moreover, in our system, no baits and no attractant such as ATP have been used as has been done in some other studies [7]. Another hypothesis to explain the attractivity of placing the plate on the cage stimulating blood feeding can be as described by Charlwood et al. [33], who showed that hungry *Ae. albopictus* females respond to recent probing and feeding by conspecifics by landing in the vicinity of sites on the host where this took place. This ‘invitation’ effect may be due to a semiochemical produced early in the feeding process. However, in our conditions, initiating several blood feedings did not seem to increase such initial attraction.

Increasing the egg-laying substrates (i.e., the number of egg cups/cage) did not lead to an increase of egg production, suggesting that in our conditions, the surface of water and egg paper was not limiting. However, there was a trend showing that three and five papers collect more eggs than just one paper. It was not statistically significant due to the variability of the results. *Ae. albopictus* is known to exhibit skip-oviposition behavior, meaning that females do not oviposit their entire batch of eggs in one location, but choose instead to oviposit a few eggs in several different sites [34,35]. Skip-oviposition behavior may ensure the greater distribution of progeny from an individual female, reduce sibling competition, and could reduce intraspecific competition by avoiding already crowded breeding sites. In our experiment, females had probably laid small clutches in every cup. For mass rearing, however, three egg-laying substrates should be offered since on every paper, as much as 5000–7000 eggs can be collected during the first two weeks. If eggs on one oviposition paper are placed in one eclosion jar, around 3000 to 5000 L1 larvae can be collected and directly transferred to one rearing tray in the larval production racks.

## 5. Conclusions

According to the results obtained and discussed, a standard laboratory rearing cage (30 × 30 × 30 cm) with 1500 adults with a ratio of 1 male to 3 females, fed one time for thirty minutes with fresh bovine blood on cellulose membranes, and with three egg cups per cage appeared to be the optimum set-up in our conditions. One cage allowed the production of 20,000 eggs during the first two weeks and between 15,000 and 10,000 during the two following weeks. According to the number of males that are required for release during pilot trials, the number of cages should be adapted, knowing that two sets of cages are needed to keep a constant high production. For example, if 20,000 males per week are needed, 40,000 male pupae need to be produced (with a male pupal productivity of 25%) [7]) per week, meaning a production of 80,000 pupae. If the pupal productivity (number of pupae produced divided by the number of eggs used) is around 80%, 100,000 eggs have to be produced per week so a maximum of 10 cages are needed. The present study shows that with optimization of *Ae. albopictus* male laboratory-scale production, small or beginner projects could develop an efficient medium-scale production with limited budget. For instance, in La Reunion island SIT project, the next pilot trials will use these parameters to be able to produce enough sterile males for the 100,000 males weekly release. Naturally, in addition, optimization of eggs, larvae, and pupae management and rearing is also needed and will be treated in further studies.
